# Supplementary material for: Systematic Review of Incidence Studies of Pneumonia in Persons with Spinal Cord Injury
Source: J Clin Med. 2021 Dec 31;11(1):211. doi: 10.3390/jcm11010211 (PMC8745509; doi:10.3390/jcm11010211)
Supplement: Supplementary file 1 [file jcm-11-00211-s001.zip › Supplementary File S1_Search Strategy.pdf]

PUBMED

Search	Query	Results
#7	#5 AND #4	40
#6	#5 AND #3	151
#5	#1 AND #2	1.199
#4	(Prevalence [mh]) OR (prevalence* [tiab]))	702.466
#3	(Incidence [mh]) OR (incidence* [tiab]))	859.324
#2	Spinal Cord Injuries [mh] OR Spinal Cord Diseases [mh] OR Paraplegia [mh] OR Quadriplegia [mh] OR Spinal Injuries [mh]) OR (spinal cord injur* [tiab] myelopathy* [tiab] OR tetraplegia* [tiab]))	165.281
#1	(Pneumonia [mh] OR Pneumonia, Ventilator-Associated [mh] OR Pneumonia, Bacterial [mh] OR Pneumonia, Aspiration [mh]) OR (pneumonia* [tiab] OR experimental lung inflammation* [tiab] OR pneumonitis [tiab] OR pneumonitides [tiab] OR pulmonary inflammation* [tiab] OR lung inflammation* [tiab] OR lobar pneumonia* [tiab] OR pneumonia, aspiration [tiab] OR ventilator-associated pneumonia [tiab] OR bacterial pneumonia* [tiab]))	219.442

EMBASE (Excerpta Medica Database)

Search	Query	Results
#31	#10 AND #24 AND #29 AND #30	439
#30	'adult'/exp OR 'adult'	8.209.639
#29	#25 OR #26 OR #27 OR #28	1.965.065
#28	'prevalence':ti OR 'prevalence':ab	786.334
#27	'prevalence'/exp Or prevalence	955.565
#26	Incidence*:ti OR incidence*:ab	996.418
#25	'incidence'/exp OR 'incidence'	1.114.538
#24	#11 OR #12 OR #13 OR #14 OR #15 OR #16 OR #17 OR #18 OR #19 OR #20 OR #21 OR #22 OR #23	363.536
#23	(traumatic AND myelopath*:ti OR traumatic) AND myelopath*:ab	499
#22	(spinal AND cord AND laceration*:ti OR spinal) AND cord AND contusion*:ab	203
#21	(spinal AND cord AND injur*:ti OR spinal) AND cord AND contusion*:ab	59.693
#20	(spinal AND cord AND contusion*:ti OR spinal) AND cord AND contusion*:ab	2.435
#19	(post AND traumatic AND myelopath*:ti OR post) AND traumatic AND myelopath*:ab	102
#18	(spinal AND cord AND trauma:ti OR spinal) AND cord AND trauma.ab	8.522
#17	myelopath*:ti OR myelopath*:ab	16.342
#16	tetraplegia:ti OR tetraplegia.ab	3.908
#15	'spinal cord injury'/exp OR 'spinal cord injury'	81.121
#14	'spine injury'exp OR 'spine injury'	53.087
#13	'spinal cord disease'/exp OR 'spinal cord disease'	271.178
#12	'paraplegia'/exp OR 'paraplegia'	34.748
#11	'quadriplegia'/exp OR quadriplegia	17.507
#10	#1 OR #2 OR #3 OR #4 OR #5 OR #6 OR #7 OR #8 OR #9	386.732
#9	Pneumonitides	24
#8	'experimental pneumonia'/exp OR 'experimental pneumonia'	456
#7	Pneumonitis:ti OR pneumonitis:ab	20.507
#6	(pulmonary AND inflammation.ti OR pulmonary) AND inflammation*.ab	41.286
#5	(('lung'/exp OR lung) AND inflammation*.ti OR 'lung'exp OR lung) AND inflammation*.ab	74.069
#4	'virus pneumonia'/exp OR 'virus pneumonia'	15.049
#3	'Bacterial pneumonia'/exp OR 'bacterial pneumonia'	31.038
#2	Pneumonia, AND (aspiration/exp OR aspiration)	23.431

#1	Pneumonia /exp OR pneumonia	319958

MEDLINE (Ovid)

Search	Query	Results
#16	10 and 15	96
#15	11 or 13	1.439.831
#14	10 and 13	20
#13	Prevalence/or prevalence.mp.	689.917
#12	10 and 11	79
#11	Incidence.mp. or incidence/	834.094
#10	8 and 9	563
#9	Pneumonia, Ventilator-Associated/ or Pneumonia/ or Pneumonia, Bacterial/ or Pneumonia virus/ or Pneumonia, Aspiration/ or Pneumonia.mp.	152.207
#8	1 or 2 or 3 or 4 or 5 or 6 or 7	90.279
#7	Myelopathy.mp.	13.065
#6	Tetraplegia.mp.	3.117
#5	Spinal Injuries.mp. or Spinal Injuries/	9.961
#4	Quadriplegia.mp. or Quadriplegia/	9.521
#3	Paraplegia/ or Paraplegia.mp.	20.106
#2	Spinal Cord Diseases.mp. of Spinal Cord Diseases/	12.724
#1	Spinal Cord Injuries.mp. or Spinal Cord Injuries/	39.109

Cochrane Central Register of Controlled Trials (CENTRAL)

Search	Query	Results
#11	#9 AND #10	0
#10	#4 OR #5 OR #6	3.111
#9	#1 OR #2 OR #3	1.062
#8	MeSH descriptor: [Incidence] explode all trees	9.454
#7	MeSH descriptor: [Cross Sectional Studies] explode all trees	4.570
#6	MeSH descriptor: [Pneumonia, Bacterial] explode all trees	730
#5	MeSH descriptor: [Pneumonia, Aspiration] explode all trees	312
#4	MeSH descriptor: [Pneumonia] explode all trees	3.111
#3	MeSH descriptor: [Spinal Injuries] explode all trees	741
#2	MeSH descriptor: [Quadriplegia] explode all trees	174
#1	MeSH descriptor: [Paraplegia] explode all trees	195

ab = abstract
exp = explode term (search under all subheadings)
MeSH = Medical Subjects Headings, The National Library of Medicine controlled vocabulary for indexing articles in PubMed
mp. = keyword search
ti = title
