# Supplementary material for: Systematic Review of Incidence Studies of Pneumonia in Persons with Spinal Cord Injury
Source: J Clin Med. 2021 Dec 31;11(1):211. doi: 10.3390/jcm11010211 (PMC8745509; doi:10.3390/jcm11010211)
Supplement: Supplementary file 1 [file jcm-11-00211-s001.zip › Supplementary File S3_Risk of Bias assessment summary.pdf]

Supplementary File S3
Summary of risk of Bias assessment.

	Was the study population representative of the target population?	Was the sampling frame a true or close representation of the target population?	Was some form of random selection used to select the sample, OR, was a census undertaken?	Was the likelihood of non-response bias minimal?	Were data collected directly from the subjects (as opposed to proxy)?	Was an acceptable case definition used in the study?	Was there a description of the study instrument that measured the parameter of interest (incidence of pneumonia)?	Was the same mode of data collection used for all subjects?	Was the length of the shortest incidence period for the parameter of interest appropriate?	Were the numerator(s) and denominator(s) for the parameter of interest appropriate?	Summary of the overall risk of study bias from the five most important items (2,4,6,7,10) regarding our study question
Choi et al. (38)	-	+	-	-	-	-	-	+	+	-	high
Citak et al. (39)	-	+	-	-	+	-	-	+	-	-	high
Fenton et al. (40)	-	+	-	-	-	+	+	+	+	-	high
Fussenich et al. (41)	-	-	-	+	-	+	+	+	+	-	high
Garcia-Leoni et al. (36)	-	+	-	-	+	+	+	+	+	-	high
Hatton et.al. (42)	-	+	-	-	-	-	-	+	+		high
Ito et al. (43)	-	+	-	-	-	-	-	+	+		high
Kamiya et al. (44)	-	+	-	-	-	+	+	+	+	-	high
McKinley et al. (45)	+	+	-	-	-	-	-	+	+	-	high
McKinley et al. (46)	-	+	+	-	-	-	-	+	+	+	high
Medee	-	+	-	-	+	-	+	+	+	+	high

et al. (37)											
Patel et al. (47)	-	-	-	-	+	-	-	+	+	-	high
Raab et al. (48)	-	+	-	+	+	-	-	+	-	-	high
Shem et al. (18)	-	+	-	+	-	-	-	+	+	-	high
Shem et al. (19)	-	+	-	-	-	-	-	+	+	-	high
Shem et al. (29)	-	+	-	-	-	-	-	+	+	-	high
Smith et al. (49)	+	+	+	-	-	-	-	+	+	-	high
Stillman et al. (50)	-	+	-	-	+	-	-	-	+	-	high
Street et al. (51)	-	+	+	-	-	-	-	-	+	-	high
Unsal-Delialioglu et al. (52)	-	+	+	+	-	-	-	+	+	-	high
Wahman et al. (53)	-	+	+	+	+	-	-	+	+	-	high
Younan et al. (54)	-	+	-	+	-	+	+	+	+	-	high
Yu et al. (55)	-	+	-	-	-	-	-	+	+	-	high
Vitaz et al. (56)	-	+	-	-	-	-	-	-	+	-	high

Two questions were modified concerning the aim of the study. The question in item 1 was rephrased to "Was the study population representative of the target population?" (26) because our systematic review included the clinical SCI population. The question in item 7 was changed into "Was there a description of the study instrument that measured the parameter of interest (incidence of pneumonia)?".

We focused on the five most important items regarding our study question, namely item 2,4,6,7,10. The worst item out of these five items was decisive for the rating of the whole study.
