# Supplementary material for: Systematic Review of Incidence Studies of Pneumonia in Persons with Spinal Cord Injury
Source: J Clin Med. 2021 Dec 31;11(1):211. doi: 10.3390/jcm11010211 (PMC8745509; doi:10.3390/jcm11010211)
Supplement: Supplementary file 1 [file jcm-11-00211-s001.zip › Supplementary File S4_List of excluded Fulltexts.pdf]

1. Aarabi B, Harrop JS, Tator CH, Alexander M, Dettori JR, Grossman RG, et al. Predictors of pulmonary complications in blunt traumatic spinal cord injury. J Neurosurg Spine. 2012;17(1 Suppl):38-45.
2. Abel R, Ruf S, Spahn B. Cervical spinal cord injury and deglutition disorders. Dysphagia. 2004;19(2):87-94.
3. Babu R, Owens TR, Thomas S, Karikari IO, Grunch BH, Moreno JR, et al. Timing of tracheostomy after anterior cervical spine fixation. J Trauma Acute Care Surg. 2013;74(4):961-6.
4. Bellemare JF, Tepas IJJ, Imami ER, Hartland L. Complications of trauma care: Risk analysis of pneumonia in 10,001 adult trauma patients. Am Surg. 1996;62(3):207-11.
5. Berrouane Y, Daudenthun I, Riegel B, Emery MN, Martin G, Krivosic R, et al. Early onset pneumonia in neurosurgical intensive care unit patients. J Hosp Infect. 1998;40(4):275-80.
6. Borrini L, Bensmail D, Thiebaut JB, Hugeron C, Rech C, Jourdan C. Occurrence of adverse events in long-term intrathecal baclofen infusion: A 1-year follow-up study of 158 adults. Arch Phys Med Rehabil. 2014;95(6):1032-8.
7. Bourassa-Moreau E, Mac-Thiong JM, Ehrmann Feldman D, Thompson C, Parent S. Complications in acute phase hospitalization of traumatic spinal cord injury: does surgical timing matter? J Trauma Acute Care Surg. 2013;74(3):849-54.
8. Brienza D, Krishnan S, Karg P, Sowa G, Allegretti AL. Predictors of pressure ulcer incidence following traumatic spinal cord injury: a secondary analysis of a prospective longitudinal study. Spinal Cord. 2018;56(1):28-34.
9. Cardenas DD, Hoffman JM, Kirshblum S, McKinley W. Etiology and incidence of rehospitalization after traumatic spinal cord injury: a multicenter analysis. Arch Phys Med Rehabil. 2004;85(11):1757-63.
10. Croce MA, Bee TK, Pritchard E, Miller PR, Fabian TC. Does optimal timing for spine fracture fixation exist? Ann Surg. 2001;233(6):851-8.
11. Croce MA, Fabian TC, Waddle-Smith L, Maxwell RA. Identification of early predictors for post-traumatic pneumonia. Am Surg. 2001;67(2):105-10.
12. Croce MA, Tolley EA, Fabian TC. A formula for prediction of posttraumatic pneumonia based on early anatomic and physiologic parameters. J Trauma. 2003;54(4):724-9.
13. Dimar JR, Fisher C, Vaccaro AR, Okonkwo DO, Dvorak M, Fehlings M, et al. Predictors of complications after spinal stabilization of thoracolumbar spine injuries. J Trauma Inj Infect Crit Care. 2010;69(6):1497-500.
14. Dokai T, Yonei T, Otsuka T, Kono R, Nagashima H. Characteristics of cervical spine injury in the aged society (the aging rate: >33.3%). Eur Spine J. 2017;26(2):S328.
15. Evans CT, Lavela SL, Weaver FM, Priebe M, Sandford P, Niemiec P et al. Epidemiology of hospital-acquired infections in veterans with spinal cord inury and disorders. Infection Control and Hospital Epidemiology 2008; 29(3): 234-242.
16. Fairshter RD, Vaziri ND, Gordon S. Frequency and spectrum of pulmonary diseases in patients with chronic renal failure associated with spinal cord injury. Respiration. 1983;44(1):58-62.
17. Fineberg SJ, Oglesby M, Patel AA, Singh K. Incidence, risk factors, and mortality associated with aspiration in cervical spine surgery. Spine (Phila Pa 1976). 2013;38(19):E1189-95.
18. Fishburn MJ, Marino RJ, Ditunno JF, Jr. Atelectasis and pneumonia in acute spinal cord injury. Arch Phys Med Rehabil. 1990;71(3):197-200.
19. Fletcher DJ, Taddonio RF, Byrne DW, Wexler LM, Cayten CG, Nealon SM, et al. Incidence of acute care complications in vertebral column fracture patients with and without spinal cord injury. Spine (Phila Pa 1976). 1995;20(10):1136-46.
20. Fraser DR, Dombrovskiy VY, Vogel TR. Infectious complications after vehicular trauma in the United States. Surgical infections. 2011;12(4):291-6.
21. Ganuza JR, Forcada AG, Gambarrutta C, Buigues EDDLL, Gonzalez VEM, Fuentes FP, et al. Effect of technique and timing of tracheostomy in patients with acute traumatic spinal cord injury undergoing mechanical ventilation. J Spinal Cord Med. 2011;34(1):76-84.
22. Gerndt SJ, Rodriguez JL, Pawlik JW, Taheri PA, Wahl WL, Micheals AJ, et al. Consequences of high-dose steroid therapy for acute spinal cord injury. J Trauma. 1997;42(2):279-84.
23. Glaesener JJ, Hasse W, Exner G, Mikschas V. [Thoracopulmonary complications of fresh fractures of the thoracic spine with neurologic damage]. Unfallchirurgie. 1992;18(5):274-9.
24. Glennie RA, Ailon T, Yang K, Batke J, Fisher CG, Dvorak MF, et al. Incidence, impact, and risk factors of adverse events in thoracic and lumbar spine fractures: an ambispective cohort analysis of 390 patients. The spine journal. 2015;15(4):629-37.
25. Göçmez C, Çelik F, Tekin R, Kamaşak K, Turan Y, Palanci Y, et al. Evaluation of risk factors affecting hospital-acquired infections in the neurosurgery intensive care unit. Int J Neurosci. 2014;124(7):503-8.

26. Grossman RG, Frankowski RF, Burau KD, Toups EG, Crommett JW, Johnson MM, et al. Incidence and severity of acute complications after spinal cord injury. J Neurosurg Spine. 2012;17(1 Suppl):119-28.
27. Hayashi T, Fujiwara Y, Sakai H, Maeda T, Ueta T, Shiba K. Risk factors for severe dysphagia in acute cervical spinal cord injury. Spinal Cord. 2017;55(10):940-3.
28. Hayashi T, Maeda T, Sakai H, Morishita Y, Shiba K. Risk factors for dysphagia in acute cervical spinal cord injury. Spine. 2015;2015:242-4.
29. Hayashi T, Sakai H, Maeda T, Shiba K. Risk factors for dysphagia in acute cervical spinal cord injury. J Orthop Res. 2016;34.
30. Hwang M, Zebracki K, Chlan KM, Vogel LC. Longitudinal changes in medical complications in adults with pediatric-onset spinal cord injury. J Spinal Cord Med. 2014;37(2):171-8.
31. Jackson AB, Groomes TE. Incidence of respiratory complications following spinal cord injury. Arch Phys Med Rehabil. 1994;75(3):270-5.
32. January AM, Zebracki K, Czworniak A, Chlan KM, Vogel LC. Predictive factors of hospitalization in adults with pediatric-onset SCI: a longitudinal analysis. Spinal Cord. 2015;53(4):314-9.
33. Kerwin AJ, Frykberg ER, Schinco MA, Griffen MM, Murphy T, Tepas JJ. The effect of early spine fixation on non-neurologic outcome. J Trauma. 2005;58(1):15-21.
34. Kerwin AJ, Yorkgitis BK, Ebler DJ, Madbak FG, Hsu AT, Crandall ML. Use of diaphragm pacing in the management of acute cervical spinal cord injury. J. Trauma Acute Care Surg. 2018;85(5):928-31.
35. Kopp MA, Watzlawick R, Martus P, Failli V, Finkenstaedt FW, Chen Y, et al. Long-term functional outcome in patients with acquired infections after acute spinal cord injury. Neurology. 2017;88(9):892-900.
36. Le Roux JC, Dunn RN. Gunshot injuries of the spine - A review of 49 cases managed at the Groote Schuur Acute Spinal Cord Injury Unit. S Afr J Surg. 2005;43(4):165-8.
37. Lehre MA, Eriksen LM, Tirsit A, Bekele S, Petros S, Park KB, et al. Outcome in patients undergoing surgery for spinal injury in an Ethiopian hospital. J Neurosurg Spine. 2015;23(6):772-9.
38. Lemons VR, Wagner Jr FC. Respiratory complications after cervical spinal cord injury. Spine. 1994;19(20):2315-20.
39. Lertudomphonwanit T, Wattanaapisit T, Chavasiri C, Chotivichit A. Risk factors relating to the need for mechanical ventilation in isolated cervical spinal cord injury patients. J. Med Assoc Thai. 2014;97:S10-S5.
40. Liu Y, Shi CG, Wang XW, Chen HJ, Wang C, Cao P, et al. Timing of surgical decompression for traumatic cervical spinal cord injury. Int Orthop. 2015;39(12):2457-63.
41. Liu XD, Wang L, Dong Z, Ma YH, Hu F, Ma GP, et al. High-dose methylprednisolone sodium succinate therapy for acute spinal cord injury in adults: A Meta-analysis. Chinese J Tissue Eng Res. 2015;19(42):6875-81.
42. MacDonald SL, Robinson LR. The Incidence of Physiatry-Relevant Complications in Trauma Patients Admitted to an Urban Canadian Trauma Center. Am J Phys Med Rehabil. 2019;98(2):165-8.
43. Marion TE, Rivers CS, Kurban D, Cheng CL, Fallah N, Batke J, et al. Previously Identified Common Post-Injury Adverse Events in Traumatic Spinal Cord Injury-Validation of Existing Literature and Relation to Selected Potentially Modifiable Comorbidities: A Prospective Canadian Cohort Study. J Neurotrauma. 2017;34(20):2883-91.
44. Martin ND, Marks JA, Donohue J, Giordano C, Cohen MJ, Weinstein MS. The mortality inflection point for age and acute cervical spinal cord injury. J Trauma. 2011;71(2):380-5.
45. McKinley WO, Jackson AB, Cardenas DD, DeVivo MJ. Long-term medical complications after traumatic spinal cord injury: a regional model systems analysis. Arch Phys Med Rehabil. 1999;80(11):1402-10.
46. Muthiah MP, Zaman MK. Mechanical ventilation simplified. South Med J. 2009;102(12):1196-7.
47. Ohbe H, Koakutsu T, Kushimoto S. Analysis of risk factors for hyponatremia in patients with acute spinal cord injury: a retrospective single-institution study in Japan. Spinal Cord. 2019;57(3):240-6.
48. Pickelsimer E, Shiroma EJ, Wilson DA. Statewide investigation of medically attended adverse health conditions of persons with spinal cord injury. J Spinal Cord Med. 2010;33(3):221-31.
49. Prabhakar MM, Rao BS, Patel L. Thoracolumbar burst fracture with complete paraplegia: Rationale for second-stage anterior decompression and fusion regarding functional outcome. J Orthop Traumatol. 2009;10(2):83-90.
50. Rose L, Leasa D, McKim D, Pedersen C, Nonoyama M, Avendano M, et al. Provision of institutional care for long-term mechanical ventilation in Canada: A national survey. Am J Respir Crit Care Med. 2014;189.

51. Samuel AM, Diaz-Collado PJ, Fu MC, Lukasiewicz AM, Webb ML, Bohl DD, et al. Hospital-acquired pneumonia occurs in 20.5% of cervical spinal cord injury patients and is associated with poor inpatient outcomes: An analysis of 5,198 patients in the national trauma data bank. Spine. 2015;2015:364-6.
52. Soden RJ, Walsh J, Middleton JW, Craven ML, Rutkowski SB, Yeo JD. Causes of death after spinal cord injury. Spinal Cord. 2000;38(10):604-10.
53. Stein DM, Menaker J, McQuillan K, Handley C, Aarabi B, Scalea TM. Risk factors for organ dysfunction and failure in patients with acute traumatic cervical spinal cord injury. Neurocritical Care. 2010;13(1):29-39.
54. Takami M, Okada M, Enyo Y, Iwasaki H, Yamada H, Yoshida M. Noncontiguous double-level unstable spinal injuries. Eur J Orthop Surg Traumatol. 2017;27(1):79-86.
55. Tan JH, Fauzi AA, Hasnan N. Pulmonary rehabilitation using mechanical insufflation-exsufflation therapy for spinal cord injury – Two case studies in the university Malaya medical centre. J. Transl. Eng. Health Med. 2017;20(2):31-3.
56. Vogel LC, Krajci KA, Anderson CJ. Adults with pediatric-onset spinal cord injury: part 1: prevalence of medical complications. J Spinal Cord Med. 2002;25(2):106-16.
57. Wall BM, Mangold T, Huch KM, Corbett C, Cooke CR. Bacteremia in the chronic spinal cord injury population: risk factors for mortality. J Spinal Cord Med. 2003;26(3):248-53.
58. Waters RL, Meyer PR, Jr., Adkins RH, Felton D. Emergency, acute, and surgical management MED SCI MONITOR of spine trauma. Arch Phys Med Rehabil. 1999;80(11):1383-90.
59. Wilson J, Harrop J, Fehlings M, Grossman R, Toups E, Aarabi B. Natural history, mortality, complications and impact of early surgical decompression in thoracic spinal cord injury: A multicenter prospective study from the North American clinical trials network and aospine spinal cord injury knowledge forum. Global Spine Journal. 2017;7(2):121S.
60. Zhao T, Yishmaan BL, Lin D, Xu K, Zhou Q, Yang G. Is Delayed Surgery After Unsuccessful Conservative Treatment Beneficial for Spinal Cord Injury Following Whiplash? A Retrospective Study in Elderly Patients. MED SCI MONITOR. 2018;24:2818-24.
61. Green BA, Green KL, Klose KJ. Kinetic therapy for spinal cord injury. Spine (Phila Pa 1976). 1983;8(7):722-8.
62. Velmahos GC, Toutouzas K, Chan L, Tillou A, Rhee P, Murray J, et al. Intubation after cervical spinal cord injury: to be done selectively or routinely? Am Surg. 2003;69(10):891-4.
63. Hachen HJ. Idealized care of the acutely injured spinal cord in Switzerland. J Trauma. 1977;17(12):931-6.
64. Hassid VJ, Schinco MA, Tepas JJ, Griffen MM, Murphy TL, Frykberg ER, et al. Definitive establishment of airway control is critical for optimal outcome in lower cervical spinal cord injury. J Trauma. 2008;65(6):1328-32.
65. Liebscher T, Niedeggen A, Estel B, Seidl RO. Airway complications in traumatic lower cervical spinal cord injury: A retrospective study. J Spinal Cord Med. 2015;38(5):607-14.
66. Romero J, Vari A, Gambarrutta C, Oliviero A. Tracheostomy timing in traumatic spinal cord injury. Eur Spine J. 2009;18(10):1452-7.
67. Choi SH, Sung CH, Heo DR, Jeong SY, Kang CN. Incidence of acute spinal cord injury and associated complications of methylprednisolone therapy: a national population-based study in South Korea. Spinal Cord. 2020;58(2):232-7.
68. MacCallum KP, Kalata S, Darcy D, Parsikia A, Chao E, Stone ME, et al. Prolonged use of spinal precautions is associated with increased morbidity in the trauma patient. Injury. 2020;51(2):317-21.
69. Hartkopp PA, Brønnum-Hansen H, Seidenschnur AM, Biering-Sørensen F. Survival and cause of death after traumatic spinal cord injury. A long-term epidemiological study. Ugeskr Laeger. 1998 Oct 19;160(43):6207-10.
70. Ragnarsson O, Geirsson G. Epidemiology and medical complications in patients with traumatic spinal cord injuries in Iceland. Laeknabladid. 1998 Jul;84(7):541-51.
71. Verschueren JH, Post MW, de Groot S, van der Woude LG, van Asbeck FW, Rol M. Occurrence and predictors of pressure ulcers during primary in-patient spinal cord injury rehabilitation. Spinal cord 2011; 49(1): 106-12.
