# Supplementary material for: Systematic Review of Incidence Studies of Pneumonia in Persons with Spinal Cord Injury
Source: J Clin Med. 2021 Dec 31;11(1):211. doi: 10.3390/jcm11010211 (PMC8745509; doi:10.3390/jcm11010211)
Supplement: Supplementary file 1 [file jcm-11-00211-s001.zip › Supplementary File S5 Sensitivity analyses with two alternative calculations of the time under risk_Revision_2.pdf]

Supplementary File S5

Table S5.A Sensitivity analyses where the time under risk for the patients with pneumonia was set to 0 (i.e. as if they had the pneumonia all at the first day).

	Univariable Meta-Regression			Multivariable Meta-Regression		
Variable	Incidence Rate Ratio	95% CI	p-value	Incidence Rate Ratio	95% CI	p-value
Setting						
Post-Rehab (Reference)	1.00			1.00		
Acute	3.39	0.68 to 16.9	0.137	0.57	0.08 to 4.16	0.578
ICU	12.11	2.63 to 55.65	0.001	2.78	0.26 to 29.29	0.394
Long-Term Ventilation	1.38	0.08 to 22.57	0.821	0.89	0.03 to 30.19	0.948
Mixed	4.53	0.28 to 73.29	0.287	22.09	1.07 to 456.06	0.045
Rehab	3.03	0.33 to 27.51	0.325	0.76	0.06 to 9.32	0.830
Level of Injury						
Persons w. Paraplegia (Reference)	1.00					
Mixed, > 50% Persons w. Paraplegia	0.32	0.01 to 8.84	0.503	0.19	0.01 to 0.01	0.367
Mixed, > 50% Persons w. Tetraplegia	2.88	0.16 to 50.73	0.470	3.58	0.29 to 0.29	0.323
Persons w. Tetraplegia	2.85	0.17 to 48.72	0.469	2.22	0.17 to 0.17	0.545
Mixed, with unknown Proportion	0.48	0.02 to 11.12	0.647	2.07	0.1 to 0.1	0.633
Ventilation						
Not mentioned (Reference)	1.00					
Mixed	6.15	2.09 to 18.12	0.001	5.93	1.46 to 24.1	0.013
Ventilated	5.24	1.79 to 15.33	0.002	1.93	0.45 to 8.27	0.374

Tracheostomy						
No (Reference)						
Mixed	3.37	1.14 to 9.95	0.028	1.32	0.39 to 4.44	0.658
Yes	3.84	1.11 to 13.35	0.034	0.48	0.09 to 2.68	0.405
Dysphagia						
No	1.00			*		
Not mentioned	1.17	0.06 to 23.53	0.918			
Mixed	1.87	0.03 to 125.92	0.771			
Yes	1.42	0.04 to 52.58	0.849			

* Too few studies with information on dysphagia (only two studies reported on dysphagia), therefore, we did not include dysphagia in the multivariable model.

Incidence Rate Ratio: exp(coefficient): how many times the incidence per 100 days is higher compared to the reference category, (in multivariable analysis: controlled for all other variables).

Table S5.B Sensitivity analyses where the time under risk for the patients with pneumonia was set to the same time as for those patients without pneumonia (i.e. as if they had the pneumonia all at the last day).

	Univariable Meta-Regression			Multivariable Meta-Regression		
Variable	Incidence Rate Ratio	95% CI	p-value	Incidence Rate Ratio	95% CI	p-value
Setting						
Post-Rehab (Reference)	1.00			1.00		
Acute	3.21	0.93 to 11.11	0.066	0.67	0.16 to 2.86	0.591
ICU	6.41	1.98 to 20.71	0.002	1.66	0.3 to 9.23	0.563
Long-Term Ventilation	1.30	0.15 to 11.09	0.813	0.84	0.07 to 10.76	0.895
Mixed	2.47	0.29 to 20.75	0.404	13.25	1.5 to 116.97	0.020
Rehab	3.36	0.62 to 18.28	0.160	0.72	0.12 to 4.37	0.720
Level of Injury						
Persons w. Paraplegia (Reference)	1.00					
Mixed, > 50% Persons w. Paraplegia	0.25	0.02 to 3.15	0.286	0.19	0.01 to 0.01	0.225
Mixed, > 50% Persons w. Tetraplegia	2.14	0.23 to 19.79	0.503	3.27	0.45 to 0.45	0.241
Persons w. Tetraplegia	1.74	0.19 to 15.84	0.621	1.94	0.26 to 0.26	0.521
Mixed, with unknown Proportion	0.49	0.04 to 5.49	0.565	1.81	0.18 to 0.18	0.613
Ventilation						
Not mentioned (Reference)	1.00					
Mixed	3.79	1.68 to 8.55	0.001	4.36	1.56 to 12.19	0.005
Ventilated	3.95	1.77 to 8.81	0.001	2.35	0.82 to 6.73	0.112

Tracheostomy							
	No (Reference)						
	Mixed	2.24	0.95 to 5.29	0.065	1.05	0.43 to 2.57	0.917
	Yes	1.82	0.68 to 4.88	0.233	0.39	0.11 to 1.35	0.136
Dysphagia							
	No	1.00			*		
	Not mentioned	0.83	0.09 to 8.05	0.874			
	Mixed	1.98	0.08 to 51.21	0.680			
	Yes	0.99	0.07 to 15.09	0.995			

* Too few studies with information on dysphagia (only two studies reported on dysphagia), therefore, we did not include dysphagia in the multivariable model.

Incidence Rate Ratio: exp(coefficient): how many times the incidence per 100 days is higher compared to the reference category, (in multivariable analysis: controlled for all other variables).
